# Comparative use of CRISPR and RNAi to modulate integrin α3β1 in triple negative breast cancer cells reveals that some pro-invasive/pro-metastatic α3β1 functions are independent of global regulation of the transcriptome

**DOI:** 10.1371/journal.pone.0254714

**Published:** 2021-07-16

**Authors:** James Kenney, Abibatou Ndoye, John M. Lamar, C. Michael DiPersio

**Affiliations:** 1 Department of Regenerative and Cancer Cell Biology, Albany Medical College, Albany, New York, United States of America; 2 Department of Surgery, Albany Medical College, Albany, New York, United States of America; 3 Department of Molecular and Cellular Physiology, Albany Medical College, Albany, New York, United States of America; University of Bergen, NORWAY

## Abstract

Integrin receptors for the extracellular matrix play critical roles at all stages of carcinogenesis, including tumor growth, tumor progression and metastasis. The laminin-binding integrin α3β1 is expressed in all epithelial tissues where it has important roles in cell survival, migration, proliferation, and gene expression programs during normal and pathological tissue remodeling. α3β1 signaling and adhesion functions promote tumor growth and metastasis in a number of different types of cancer cells. Previously, we used RNA interference (RNAi) technology to suppress the expression of the *ITGA3* gene (encoding the α3 subunit) in the triple-negative breast cancer cell line, MDA-MB-231, thereby generating variants of this line with reduced expression of integrin α3β1. This approach revealed that α3β1 promotes pro-tumorigenic functions such as cell invasion, lung metastasis, and gene regulation. In the current study, we used CRISPR technology to knock out the *ITGA3* gene in MDA-MB-231 cells, thereby ablating expression of integrin α3β1 entirely. RNA-seq analysis revealed that while the global transcriptome was altered substantially by RNAi-mediated suppression of α3β1, it was largely unaffected following CRISPR-mediated ablation of α3β1. Moreover, restoring α3β1 to the latter cells through inducible expression of α3 cDNA failed to alter gene expression substantially, suggesting that use of CRISPR to abolish α3β1 led to a decoupling of the integrin from its ability to regulate the transcriptome. Interestingly, both cell invasion *in vitro* and metastatic colonization *in vivo* were reduced when α3β1 was abolished using CRISPR, as we observed previously using RNAi to suppress α3β1. Taken together, our results show that pro-invasive/pro-metastatic roles for α3β1 are not dependent on its ability to regulate the transcriptome. Moreover, our finding that use of RNAi versus CRISPR to target α3β1 produced distinct effects on gene expression underlines the importance of using multiple approaches to obtain a complete picture of an integrin’s functions in cancer cells.

## Introduction

Integrins are heterodimeric, transmembrane proteins consisting of an α and a β subunit that function as the major cell surface receptors for cell adhesion to the extracellular matrix (ECM) [1]. In addition to providing a physical linkage between the ECM outside the cell and the cytoskeleton inside the cell, integrins function as conduits of bidirectional signal transduction that allows cells to both modify and respond to cues from the tissue microenvironment [1]. There are 24 distinct integrins with different ligand binding specificities and signaling functions [1, 2]. Genetic studies using mouse knockout models have revealed distinct phenotypes caused by the deletion of the genes that encode individual α or β subunits, indicating that different integrins have non-redundant, although sometimes overlapping functions [2–4]. Integrins are important at every stage of cancer progression and metastasis [5], and their normal functions are deregulated in many types of cancer cells [6], including breast cancer cells [7].

One of the most common genetic approaches to identify functions of a specific integrin is use of RNA interference (RNAi) to suppress the expression of either the α or β subunit, resulting in reduced cell surface expression of the αβ heterodimer [8–14]. RNAi is widely used to suppress or “knock down” the expression of a target gene through transient expression of a small interfering RNA (siRNA), or through stable expression of a short hairpin RNA (shRNA), that is designed to neutralize the target mRNA transcript and inhibit gene expression or translation [15]. Since some siRNAs/shRNAs can produce off-target effects that may obfuscate the target gene’s function, inclusion of a non-targeting siRNA/shRNA as an experimental control is essential [15, 16].

In recent years, CRISPR (Clustered Regularly Interspaced Short Palindromic Repeats) has emerged as a powerful approach to edit the mammalian genome, including modulation of a target gene’s expression [17, 18]. Although this newer technology has been used less extensively than RNAi to investigate integrins, CRISPR-mediated ablation of integrin expression has been applied in different contexts [19–25]. This approach exploits the ability of the CRISPR-associated protein 9 (Cas9) endonuclease to be targeted to a specific gene when complexed with a CRISPR RNA (crRNA) that is complementary to a DNA sequence within the gene [17]. Cas9-mediated DNA cleavage leaves a double-strand break that is repaired through non-homologous end joining, which can introduce a frame shift mutation that ablates expression of the encoded protein [17]. A caveat of CRISPR is the potential for off-target mutations in other genes [26].

When investigating an integrin gene, it seems reasonable to predict that use of either CRISPR to ablate its expression or RNAi to suppress its expression may lead to similar phenotypes. However, an important caveat is that RNAi usually causes reduced expression of the target gene, while CRISPR may lead to its complete ablation. The laminin-binding integrin α3β1 promotes tumor growth, invasion, and metastasis of breast cancer cells, although this role may be context dependent [27–29]. Studies using RNAi to knockdown the α3 integrin subunit in breast cancer cells have identified α3β1-dependent gene regulation that promotes tumor cell growth and invasion [11–13]. In the current study, we used CRISPR to target the *ITGA3* gene that encodes the α3 integrin subunit in MDA-MB-231 cells, a widely used model of triple-negative breast cancer (TNBC), thereby generating a variant line in which expression of α3β1 is entirely absent (hereafter referred to as α3-Cr cells). In parallel, we used dicer-substrate siRNA (dsiRNA) to suppress *ITGA3* mRNA in MDA-MB-231 cells, which led to substantial but incomplete loss of α3β1. RNA-seq analysis revealed strikingly different effects of these two approaches on the transcriptome, where RNAi-targeting of *ITGA3* caused changes in 883 genes, while CRISPR-targeting of *ITGA3* caused changes in an overlapping, but largely distinct set of only 37 genes. Moreover, rescue of α3β1 expression in α3-Cr cells using a doxycycline-inducible α3 model had a minimal effect on the transcriptome, suggesting that use of CRISPR to eliminate α3β1 led to a cellular adaptation such that gene regulation was no longer responsive to the integrin. Interestingly, α3-Cr cells showed reduced cell invasion *in vitro* and impaired metastatic colonization *in vivo* compared with control cells, as we reported previously for RNAi-mediated suppression of α3β1 [12, 13]. Our results identify a requirement for α3β1 in invasion and metastasis that is independent of its ability to regulate gene expression on a global scale. Our findings also highlight the importance of using more than one genetic approach to identify the full range of cellular functions that are regulated by an integrin.

## Results

### CRISPR-mediated ablation of α3β1 and RNAi-mediated suppression of α3β1 produce distinct effects on the transcriptome of MDA-MB-231 cells

**Fig 1:**
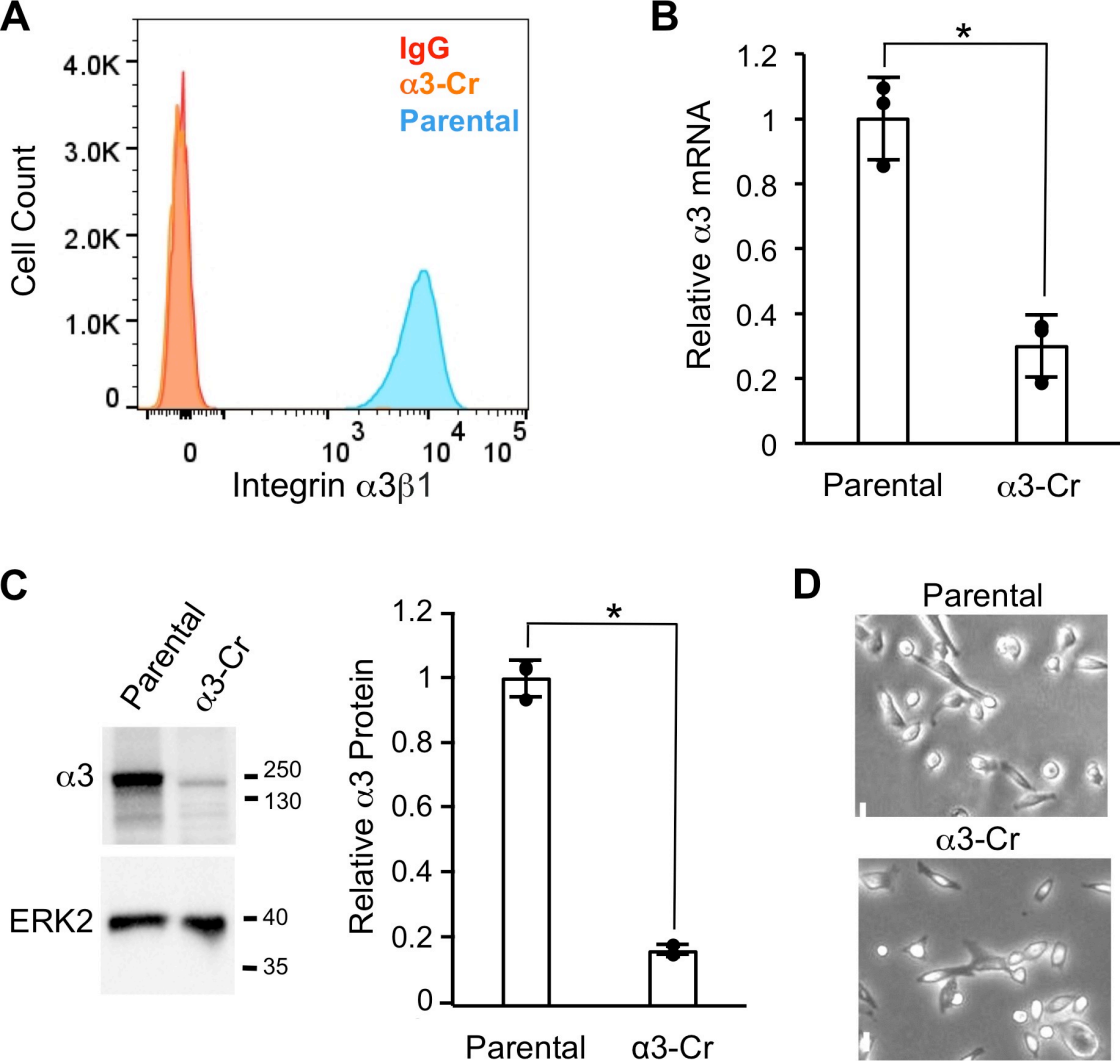
CRISPR-targeting of the *ITGA3* gene leads to loss of integrin α3β1 expression. (A) Flow cytometry with P1B5, an anti-α3 integrin monoclonal antibody, to compare surface levels of α3β1 in parental MDA-MB-231 cells (blue peak) and CRISPR-generated α3-Cr cells (orange peak). Note that the peak for α3-Cr cells overlaps with that for parental cells assayed with IgG as a negative control (red peak). (B) qPCR to assess α3 mRNA levels in α3-Cr cells relative to parental cells; *n* = 3; mean +/− SD; *p < 0.05, two-tailed t-test. (C) Western blot analysis to assess α3 protein in α3-Cr cells compared to parental cells; *ERK2*, loading control; molecular weight markers are indicated (kDa). Graph shows quantification of relative α3 protein, normalized to ERK2; *n* = 3; mean +/− SD; *p < 0.05, two-tailed t-test. (D) Bright field images of parental and α3-Cr cells. Scale bar, 10 μM.

To build from our previous investigations of integrin α3β1 in promoting pro-tumorigenic functions of TNBC cells [11–13], we used CRISPR to target the *ITGA3* gene (which encodes the α3 integrin subunit) within the MDA-MB-231 cell line. As α3 is known to partner exclusively with the β1 integrin subunit [1], this strategy was employed to generate cells in which expression of α3β1 is completely ablated (i.e., α3-Cr cells). MDA-MB-231 cells were transfected with purified Cas9 protein complexed with a crRNA that targets exon 1 of the *ITGA3* gene. The population was then sorted by flow cytometry using an anti-α3 monoclonal antibody to enrich for cells that lack α3β1 on the cell surface. Flow cytometry of this sorted population confirmed that the α3β1 heterodimer was ablated from the surface of 97% of the α3-Cr cells (Fig 1A). Additionally, *ITGA3* mRNA was reduced ~10-fold in this sorted population as demonstrated by qPCR (Fig 1B), presumably reflecting destabilization of the mRNA transcript, and α3 protein was reduced as assessed by western blot (Fig 1C). The morphology of α3-Cr cells appeared similar to that of parental MDA-MB-231 cells under standard culture conditions (Fig 1C).

**Fig 2:**
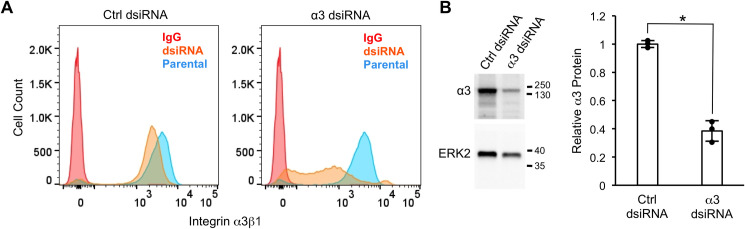
Suppression of the *ITGA3* gene using dsiRNA leads to reduced cell surface levels of integrin α3β1. MDA-MB-231 cells were transfected with control (Ctrl dsiRNA) or α3-targeting dicer substrate siRNA (α3 dsiRNA), then assayed by flow cytometry with monoclonal antibody P1B5 to compare surface levels of α3β1 (A), or by western blot to compare α3 protein expression (B). (A) Graphs show data for cells transfected with control dsiRNA (orange peak, left graph) or α3-targeting dsiRNA (orange peak, right graph). Flow cytometry was performed on parental cells with monoclonal antibody P1B5 as a positive control (shared blue peak for both graphs), or with IgG as a negative control (shared red peak for both graphs). (B) Western blot analysis to compare α3 protein in cells treated with control dsiRNA or α3-targeting dsiRNA; *ERK2*, loading control; molecular weight markers are indicated (kDa). Graph shows quantification of relative α3 protein, normalized to ERK2; *n* = 3; mean +/− SD; *p < 0.05, two-tailed t-test.

In a parallel approach, we used dsiRNA to suppress *ITGA3* mRNA. MDA-MB-231 cells were reverse-transfected with control or α3-targeting dsiRNA then cultured for 4 days under standard growth conditions prior to flow cytometry to assess cell surface levels of α3β1. Treatment with α3-targeting dsiRNA led to substantially reduced α3β1 levels on the cell surface assessed by flow cytometry (Fig 2A), as well as reduced α3 protein assessed by western blot (Fig 2B). The partial suppression of cell surface α3β1 using dsiRNA was distinct from the complete absence of cell surface α3β1 that we had observed following CRISPR-mediated ablation of *ITGA3* in α3-Cr cells (see Fig 1A).

**Fig 3:**
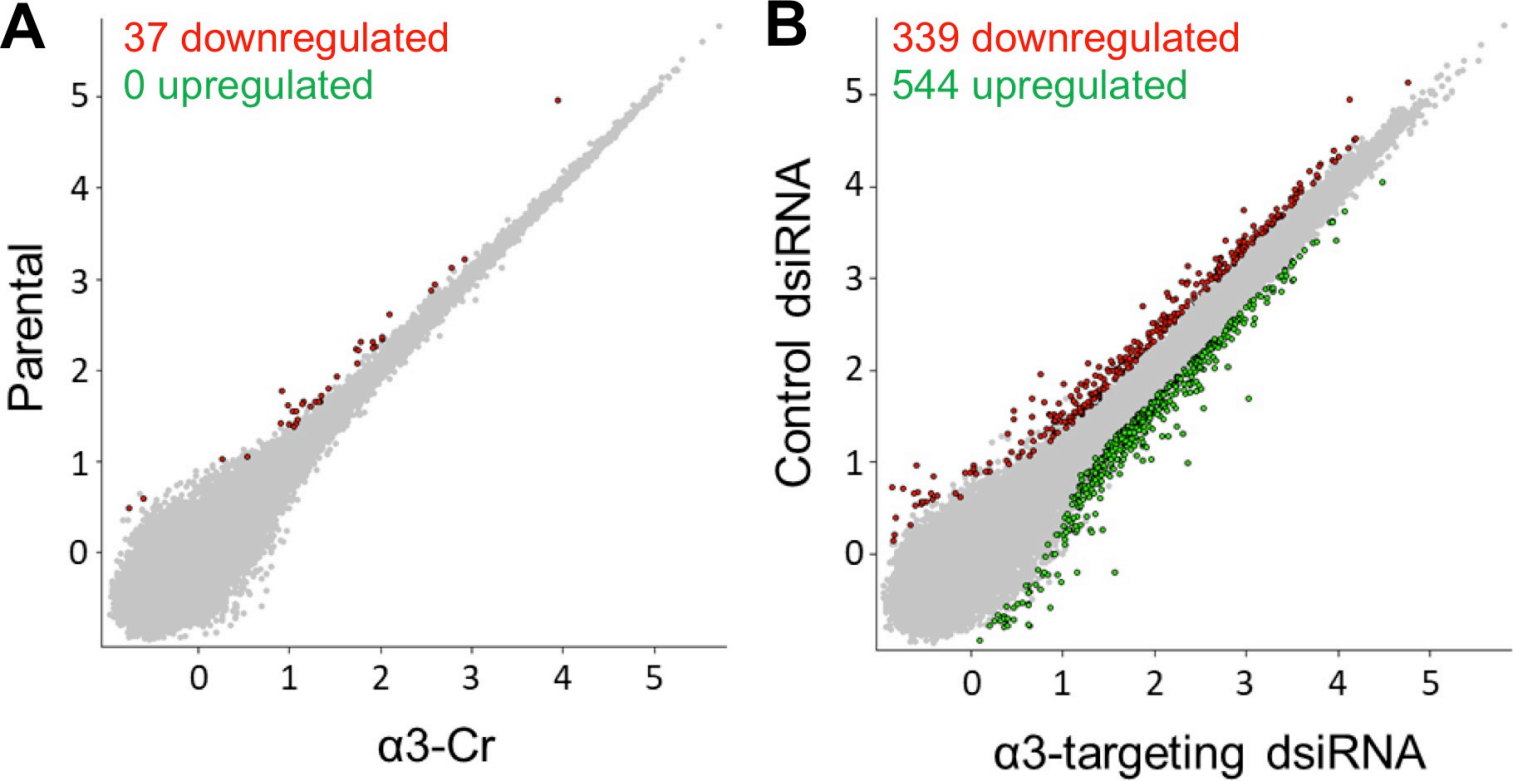
CRISPR-targeting and RNAi-targeting of the *ITGA3* gene have distinct effects on the MDA-MB-231 cell transcriptome. Scatterplots (relative gene expression, log_10_ scale) show pairwise comparisons of RNA-seq data from (A) parental MBA-MB-231 cells vs. CRISPR-generated α3-Cr cells, or from (B) MBA-MB-231 cells treated with control dsiRNA vs. α3-targeting dsiRNA. The number of genes that were down-regulated (red) or up-regulated (green) in ITGA3-targeted cells, from a total of 37,671 discovered genes, is indicated. False discovery rate (FDR) < 0.05; fold-change > 2.0.

We reasoned that CRISPR-mediated α3 ablation may alter the transcriptome more extensively than RNAi-mediated α3 suppression, as some α3β1-regulated genes may be responsive to residual levels of α3β1 that remain in cells treated with α3-targeting dsiRNA. To test this hypothesis, we carried out comparative RNA-seq analysis of parental MDA-MB-231 cells versus α3-Cr cells, and of cells transfected with control versus α3-targeting dsiRNA (described above). To our surprise, scatterplot analysis of RNA-seq data revealed that effects on the transcriptome were very different between the two approaches. Indeed, only 37 genes were altered in α3-Cr cells relative to parental cells (Fig 3A). In striking contrast, 339 genes showed lower expression, and 544 genes showed higher expression, in cells transfected with α3-targeting dsiRNA compared with control dsiRNA (Fig 3B). Thus, gene expression was altered much more dramatically by RNAi-mediated suppression of α3β1 than by CRISPR-mediated ablation of α3β1. These transcriptomic differences suggest that there is a threshold level of α3β1 below which the integrin no longer regulates gene expression. Alternatively, RNAi may induce cellular stress that activates α3β1-dependent gene programs, while use of CRISPR does not.

Table 1 lists the 37 genes that showed reduced expression in α3-Cr cells compared with parental cells (no genes showed increased expression), as well as the top 40 genes that showed either reduced or increased expression in α3 dsiRNA-treated cells compared with control dsiRNA-treated cells. Gene ontology analysis of RNA-seq data from the dsiRNA condition (GENEWIZ) revealed an enrichment of α3β1-regulated genes involved in the regulation of gene expression (GO:0010628) and cell migration (GO:0030335), both of which are integrin α3β1 functions than may contribute to cell invasion/metastasis [27, 30]. However, of the 37 genes that were down-regulated in α3-Cr cells, only 10 were also down-regulated when α3 was suppressed using dsiRNA (these genes are indicated with an asterisk in Table 1). Of these overlapping genes, several have been implicated in cancer migration/invasion or metastasis, including EPHB6 [31], AGR2 [32], RAB37 [33], CST1 [34], and B4GALNT3 [35]. PTGS2, which encodes cyclooxygenase-2, is of particular interest since we showed previously in MDA-MB-231 cells that it is regulated by α3β1 and promotes invasion [13].

**Table 1:** Differentially expressed genes in cells with CRISPR-mediated ablation or RNAi-mediated suppression of *ITGA3*.

CRISPR-mediated ablation of α3		RNAi-mediated suppression of α3			
reduced expression	fold-difference	reduced expression	fold-difference	increased expression	fold-difference
AC079298.3	18.09	AQP5	7.16	SCG2	21.57
AC063976.2	15.50	ITGA3	6.68	PPM1E	10.00
*ITGA3	10.30	PRSS2	5.85	TMEM198	9.02
*AC002401.4	7.24	IL24	5.79	BRSK2	8.57
PSMD10P2	5.89	PRRG4	5.10	NRCAM	7.17
ADGRF4	4.28	IL6	4.61	CPLX1	6.34
*EPHB6	3.41	FRRS1	4.48	PSG4	5.81
*PRSS2	3.35	FPR1	4.47	CDK5R2	5.12
ACTBL2	3.35	LRATD2	4.38	DIO2	4.78
KRT17	3.32	RSAD2	3.95	LAMA4	4.44
GLDN	3.30	ST14	3.82	ATP1A3	4.28
DIRAS3	3.24	NIPSNAP3A	3.78	PODXL2	3.78
*AGR2	3.19	MYH15	3.71	AC087501.4	3.77
*RAB37	3.17	DMBT1	3.51	PANX2	3.77
FAM110B	3.02	TNFSF15	3.46	PPFIA3	3.73
SDR16C5	2.93	FAXDC2	3.37	SLC7A11	3.71
FRK	2.65	TTC7B	3.29	KLF15	3.46
MYO1F	2.59	SPNS2	3.28	EEF1A2	3.42
DSC2	2.55	C4orf3	3.11	DISP2	3.41
PLEKHG6	2.40	RHOC	3.06	HMOX1	3.38
*PLAAT5	2.38	MZT2B	3.03	NAT8L	3.36
*B4GALNT3	2.36	SUSD1	3.02	CCDC190	3.35
OAS1	2.33	ATXN1L	2.90	PPP1R14B-AS1	3.30
MOB3B	2.32	PLAT	2.89	MUSK	3.19
CLIC2	2.24	SQOR	2.81	GCKR	3.14
MISP	2.22	TBPL1	2.75	ATOH8	3.13
KCNS3	2.22	HNRNPUL1	2.75	IFITM1	3.07
*TNS4	2.20	S100A4	2.74	AC245041.1	3.06
*PTGS2	2.18	GJB2	2.74	TAGLN	3.00
CHN2	2.17	GPX3	2.72	GREB1L	2.86
PCDH7	2.16	PDLIM2	2.70	PCOLCE2	2.83
TSPAN8	2.15	GNG12	2.69	RTN2	2.72
MMP1	2.15	SLC22A5	2.69	PCSK1N	2.71
TSPAN1	2.12	AGK	2.67	H2BC5	2.71
MCPH1-AS1	2.10	DUSP7	2.66	SPTBN2	2.68
MAL2	2.08	SH2D3A	2.56	SMARCD3	2.62
*CST1	2.00	STX17	2.55	FAM171A2	2.57
---	---	THBD	2.50	SPHK1	2.51
---	---	GPR68	2.47	LINC00472	2.51
---	---	SMAGP	2.47	AC245041.2	2.48

For CRISPR-modified cells, all 37 genes are listed that showed reduced expression in α3-Cr cells compared with parental cells. For α3 dsiRNA-treated cells, the top 40 genes are listed that showed either reduced or increased expression compared with control dsiRNA-treated cells.

*, asterisks indicate genes in the CRISPR-modified cells that were also reduced in α3 dsiRNA-treated cells (note that some of these genes are not among the top listed genes in the latter group). False discovery rate (FDR) < 0.05; fold-change > 2.0. Full data sets have been deposited in the Harvard Dataverse (https://dataverse.harvard.edu/; doi, https://doi.org/10.7910/DVN/D9G2GQ).

### Restoration of α3β1 to α3-Cr cells fails to restore α3β1-responsive gene expression

**Fig 4:**
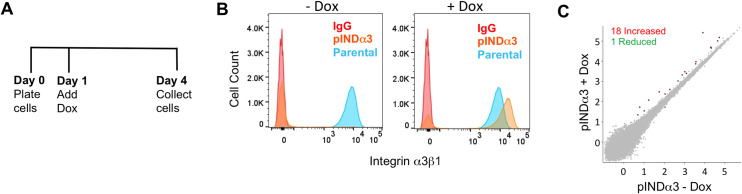
Restoration of α3β1 to α3-Cr cells fails to restore α3β1-regulated gene expression. (A) Timeline of pINDα3 cells treatment with doxycycline to induce α3β1 expression prior to flow cytometry and RNA-seq analysis. (B) Flow cytometry with monoclonal antibody P1B5 to assess surface levels of α3β1 in pINDα3 cells (orange peaks) that were left untreated (-Dox, left graph) or were treated with doxycycline (+Dox, right graph). Parental cells were assayed with P1B5 as a positive control (shared blue peak for both graphs), or with IgG as a negative control (shared red peak for both graphs). (C) Scatterplot (relative gene expression, log_10_ scale) shows pairwise comparison of RNA-seq data from pINDα3 cells that were either untreated or treated with doxycycline. The number of genes that showed increased expression (red) or reduced expression (green) in doxycycline-treated cells (i.e., with restored α3β1 expression) compared to untreated, from a total of 37,671 discovered genes, is indicated. FDR < 0.05; fold-change > 2.0.

The above findings suggested that gene regulation is somehow rendered less responsive to α3β1 in α3-Cr cells. To test this hypothesis directly, we restored α3β1 expression in α3-Cr cells, then performed RNA-seq to assess effects on the transcriptome. We transduced α3-Cr cells with a modified pINDUCER20 lentivirus in which human α3 cDNA expression is under control of a doxycycline-inducible, minimal CMV promoter to generate pINDα3 cells. pINDα3 cells were seeded at equal numbers, then left untreated or treated with doxycycline for three days prior to isolation of RNA for RNA-seq (Fig 4A). Flow cytometry of an aliquot of cells prior to RNA isolation confirmed that α3β1 was undetectable on the surface of untreated pINDα3 cells, and that doxycycline treatment induced α3β1 in the majority of cells to a level that was comparable to that on parental cells (Fig 4B). Remarkably, RNA-seq revealed only 19 genes with significantly different expression (1 up-regulated, 18 down-regulated) following doxycycline-treatment of pINDα3 cells (Fig 4C). With the exception of ITGA3 itself, there was no overlap between these 19 genes and the 37 genes that were altered in α3-Cr cells compared with parental cells (Fig 3A). This minimal change in gene expression upon induction of α3β1 is in striking contrast with the changes that we had observed in 883 genes following dsiRNA-mediated suppression of α3 (see Fig 3B). These results suggest that use of CRISPR to abolish α3β1 could decouple the integrin from its gene regulatory functions, possibly reflecting an adaptive response in these cells that maintains gene expression that is normally dependent upon α3β1.

### α3β1 promotes breast cancer cell invasion and metastatic colonization independently of global regulation of the transcriptome

**Fig 5:**
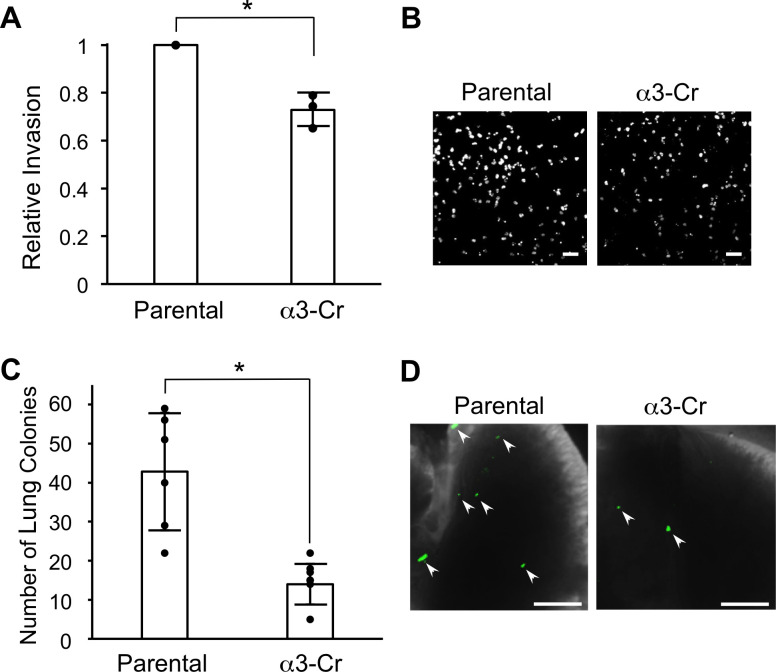
CRISPR-targeting of the *ITGA3* gene reduces cell invasion *in vitro* and metastatic colonization *in vivo*. Matrigel invasion assays were performed to compare invasion in parental MDA-MB-231 cells and α3-Cr cells. (A) Graph shows α3-Cr cell invasion relative to parental. Nuclei were stained with DAPI, imaged and quantified; *n* = 3; mean +/− SD; *p < 0.05, unpaired t-test. (B) Images show representative fields of DAPI-stained cells that invaded through transwell filters. Scale bars,100 μm. (C) Parental MDA-MB-231 cells or α3-Cr cells were labeled fluorescently by transduction with a lentivirus expressing ZsGreen, then 1 X 10^4^ cells were injected into tail-veins of 5-week old, female NSG™ mice. Graph shows number of lung colonies 21 days post-injection; *n* = 6 (Parental) or 7 (α3-Cr); mean +/- SD; *p < 0.05; two-tailed t-test. (D) Images show portions of lungs at time of harvest. Arrowheads point to examples of colonies detected by green fluorescence. Scale bars, 2 mm.

Previously, we showed that RNAi-mediated suppression of *ITGA3* in MDA-MB-231 cells substantially reduced cell invasion *in vitro* and metastatic lung colonization *in vivo*, and we have shown that these phenotypes are due at least partly to the loss of α3β1-dependent gene regulation in these cells [11–13]. However, α3β1 also has important roles in cell adhesion and migration, raising the possibility that this integrin also promotes invasion and/or metastasis independently of its ability to regulate gene expression. Since CRISPR-mediated ablation of α3β1 did not lead to extensive changes in the transcriptome, we used α3-Cr cells to assess whether α3β1 can regulate invasion and metastatic colonization in the absence of such changes. Using a Matrigel transwell assay, we observed significantly reduced invasion of α3-Cr cells compared with parental MDA-MB-231 cells (Fig 5A and 5B). To determine whether CRISPR-mediated ablation of α3β1 alters metastatic colonization *in vivo*, parental or α3-Cr cells were labeled fluorescently through transduction with a lentivirus expressing ZsGreen and then injected into the tail veins of NSG™ mice, and lungs were harvested after 21 days to assess metastatic colonies. Lungs from mice injected with α3-Cr cells showed a ~3-fold decrease in metastatic colonies compared with lungs from mice injected with parental cells (Fig 5C and 5D), similar to the decrease that we reported previously using RNAi to suppress α3 [12]. These findings demonstrate that α3β1 promotes invasion and metastatic colonization independently of its ability to regulate global gene expression.

## Discussion

Genetic approaches to manipulate the expression of genes that encode individual α or β integrin subunits have been instrumental in defining the roles that different integrin heterodimers play in normal and pathological processes, including cancer. While RNAi and CRISPR have each been applied to the investigation of integrins (see Introduction), studies are lacking that directly compare resulting phenotypes when these two approaches are used to manipulate expression of the same integrin. Our current findings reveal both similarities and distinctions in the phenotypes produced when using RNAi versus CRISPR to modify the expression of integrin α3β1 in TNBC cells. Indeed, effects on the transcriptome were remarkably different between the two approaches, as gene expression was altered dramatically by RNAi-mediated suppression of α3β1 but far less so by CRISPR-mediated ablation of α3β1. On the other hand, invasion and metastatic colonization were both reduced when CRISPR was used to eliminate α3β1, as we reported previously for RNAi-mediated suppression of α3β1 [11–13].

Although our previous studies using RNAi showed that α3β1-dependent gene regulation contributes to invasion and metastasis [11–13], here we show that α3β1 also promotes these functions independently of global changes in the transcriptome, most likely due to loss of ECM-binding functions following CRISPR-mediated deletion of α3. We do not yet know whether reduced invasion and metastatic colonization observed in α3-Cr cells is due to absence of α3β1-mediated traction force during migration, and/or to absence of α3β1-dependent signaling pathways that may not require gene expression changes, such as anoikis-resistance signaling [36]. In any case, our findings reveal the limitations of relying solely on transcriptomic approaches to determine how α3β1 controls invasion/metastasis, or to understand the mechanistic underpinnings of phenotypic differences between α3β1-deficient cells generated using siRNA or CRISPR. Future studies to assess global changes in protein expression (e.g., proteomics) or post-translational modification/signaling (e.g., phosphoproteomics) may provide insights into why some α3β1-regulated cell functions are lost from α3-Cr cells (e.g., invasion and metastatic colonization), while others are retained (e.g., transcriptome expression).

Our observation that α3-Cr cells did not show substantial transcriptome changes when α3β1 was restored in a doxycycline-inducible rescue model indicates that these cells have lost responsiveness to α3β1 with regard to gene regulation. This finding suggests that CRISPR-mediated ablation of α3β1 somehow leads to a cellular adaptation wherein the integrin is irreversibly decoupled from gene regulation, while RNAi-mediated suppression of α3β1 does not. Such an adaptation could involve a compensatory mechanism that is activated to maintain gene expression when α3β1 is entirely ablated (i.e., using CRISPR). This adaptation may not occur in the presence of residual α3β1 (i.e., using RNAi), perhaps because there is greater selective pressure in the absence of the integrin to activate such mechanisms. Support for adaptive responses following genetic ablation of α3β1 includes a study from the Sonnenberg group, which showed that α3-null epidermis formed fewer skin tumors in a chemical carcinogenesis model, but those α3β1-deficient tumors that did grow showed more rapid progression to carcinoma [37]. Another possibility is that use of RNAi induces cellular stress that activates α3β1-dependent gene programs, while use of CRISPR does not. In any case, our findings highlight the different phenotypes that are obtained using CRISPR versus RNAi to modulate α3β1, which could extend to studies of other integrins.

Although the mechanistic underpinnings of our discrepant findings using RNAi versus CRISPR to assess α3β1-dependent gene regulation remain unclear, it is unlikely that this discrepancy resulted simply from off-target or non-specific effects of CRISPR. Indeed, the possibility that such an effect would maintain the regulation of several hundred α3β1-responsive genes in α3-Cr cells seems remote. Rather, we propose that there is an important distinction between using RNAi to incompletely suppress an integrin, and using CRISPR to completely ablate an integrin. RNAi rarely eliminates the expression of a target gene, and knockdown efficiencies of distinct siRNAs can vary considerably with suppression to a level of <80% being common [38]. Indeed, RNAi targeting of the *ITGA3* mRNA transcript leads to its partial suppression, leaving residual α3β1 on the cell surface (Fig 2) [13]. In contrast, CRISPR-mediated ablation of the *ITGA3* gene resulted in the complete loss of α3β1 from the cell surface (Fig 1A). Thus, it is possible that the degree to which expression of α3β1 is reduced influences its gene regulatory functions, such that these functions are lost when its cell surface level drops below a certain threshold. Although this hypothesis remains to be tested, such an effect is reminiscent of dose effects that have been reported for RGD-mimetic integrin inhibitors [39], or following genetic or pharmacological inhibition of integrin signaling effectors such as focal adhesion kinase [40]. Importantly, this consideration may apply broadly to approaches that result in only partial suppression of a targeted integrin’s expression or function (e.g., RNAi, morpholinos, function-blocking antibodies or peptides) versus those that completely eliminate the expression of the integrin (e.g., CRISPR, gene knockout).

It is possible that compensatory mechanisms that can maintain α3β1-dependent phenotypes are mediated by other integrins with overlapping function that are activated following CRISPR-mediated deletion of α3, but not following RNAi-mediated suppression of α3. Gene knockout mouse models have revealed that while different integrins have distinct roles in developmental and post-developmental processes [3], some integrins do have overlapping functions. Such functional overlap provides opportunity for compensation by another integrin that may hide some roles for an integrin that is targeted for genetic deletion. Indeed, it is well known that suppression/deletion of an integrin can sometimes lead to compensation by other integrins with overlapping function [41–45]. This phenomenon, sometimes referred to as “integrin switching” [45, 46], makes it important to consider whether or not such compensation occurs when a particular genetic manipulation is used to modify a target integrin. Future studies will investigate the possibility that other integrins are able to compensate to maintain gene expression in α3β1-deficient α3-Cr cells, as there is precedent for such compensation in other α3β1-deficient models [43, 44].

In summary, our unexpected finding that use of RNAi and CRISPR to target integrin α3β1 produced overlapping, but also distinct cellular phenotypes highlights the importance of considering specific limitations of different genetic approaches, and of using multiple approaches to provide a complete picture of an integrin’s roles in a particular cell type or process. Our findings also have potential implications regarding the development of α3β1 (and perhaps of integrins in general) as therapeutic targets for the treatment of cancer or other pathologies [27]. For example, if compensatory mechanisms are activated to restore α3β1-dependent gene regulation only when the integrin is inhibited below a certain threshold, then the extent to which α3β1 is therapeutically inhibited may influence clinical outcomes. Indeed, the dose-dependent effect of RGD-mimetic integrin inhibitors on tumor growth and angiogenesis has been documented in preclinical cancer models [39] and may impact strategies for clinical application of such inhibitors [47]. Future investigations using *in vivo* breast cancer models will include assessing whether partial versus complete α3β1 inhibition has distinct effects at different stages of tumor growth and progression.

## Materials and methods

### Cell culture

MDA-MB-231 cells (American Type Culture Collection, ATCC, Manassas, VA) were cultured at 37°C, 5% CO_2_ in Dulbecco’s Modified medium (DMEM) (Corning, Waltham, MA) supplemented with 10% fetal bovine serum (Gemini Bio-Products, West Sacramento, CA) and 1% L-glutamine (Gibco, Waltham, MA).

### CRISPR knockout of ITGA3

MBA-MB-231 cells with knockout of the *ITGA3* gene (α3-Cr cells) were generated with the Alt-R CRISPR-Cas9 System (Integrated DNA Technologies, IDT, Coralville, Iowa) using a mix of a predesigned CRISPR RNA (crRNA) that is specific for exon 1 sequences in the *ITGA3* gene (Hs.Cas9.ITGA3.1.AA; GATGGCTACACCAACCGGAC; IDT), a conserved transactivating crRNA (tracrRNA), and Alt-R S. *pyogenes* Cas9 nuclease. crRNA and tracrRNA were mixed together (1 μM each), heated for 5 min at 95 °C, then cooled to room temperature. Alt-R Cas9 was added (1 μM) and incubated at room temperature for 5 min. Lipofectamine RNAiMax transfection reagent (Invitrogen, Waltham, MA) was then added and the mixture was incubated for 20 min at room temperature. Reverse transfected mixture was added to 320,000 cells in 6-well plates, to achieve a concentration of 10 nM ribonucleoprotein complex. After two days of culture, efficiency of α3 knockout was determined by flow cytometry (see below), and α3-negative cells were enriched by fluorescence activated cell sorting.

### siRNA

Cells were transfected using RNAiMax (cat. 13778100, Invitrogen) following the manufacturer’s protocols. Briefly, a final concentration of 10 nM dicer-substrate non-targeting control (cat. 51-01-14-03; IDT) or α3-targeting siRNA (cat. hs.Ri.ITGA3.13.2; IDT) were incubated with RNAiMax diluted in OptiMEM (cat. 31985070 Gibco). For RNA-seq, 400,000 cells were seeded onto tissue culture treated 15 cm plates, medium was changed after 72 hours, and cells were harvested 96 hours later for RNA isolation. For western blots, 300,000 cells were seeded onto tissue culture treated 6-well plates, medium was changed after 48 hours, and cells were harvested 72 hours later for preparation of lysates.

### Western blotting

Whole cell lysates were prepared in non-reducing lysis buffer (cat. 9803, Cell Signaling, Waltham, MA) supplemented with protease inhibitor (cat. 11836170001, Roche, St. Louis, MO), 0.1% Sodium dodecyl sulfate, and 0.5% sodium deoxycholate. Samples were run on 10% SDS-PAGE, transferred to nitrocellulose membranes, and blocked in 5% bovine serum albumin. Membranes were incubated overnight at 4°C with rabbit polyclonal antibodies against integrin α3 [48] or ERK2 (cat. sc-154, Santa Cruz, Dallas, TX), then incubated at room temperature for 1 hour with HRP-crosslinked goat anti-rabbit (cat. 7074, Cell signaling), or goat anti-mouse (Cat. 62–6520, ThermoFisher, Waltham, MA). Blots were treated with Clarity Western ECL substrate (1705060, Bio-Rad, Hercules, CA) then imaged and analyzed using Image Lab software (Bio-Rad).

### RNA isolation and qPCR

Parental and α3-Cr cells were cultured for 4 days under standard growth conditions prior to RNA isolation for RNA-seq. dsiRNA-treated cells were reverse-transfected with control or α3-targeting dsiRNA then cultured for 4 days under standard growth conditions prior to RNA isolation. To confirm ablation or suppression of α3β1, flow cytometry was performed as described below on an aliquot of the same cells analyzed by RNA-seq. RNA was isolated using Trizol Reagent (Life Technologies, Waltham, MA) according to the manufacturer’s protocol, then DNAse treated using Turbo DNA-free™ Kit (Ambion, Waltham, MA). RNA quality was assessed using a NanoDrop 1000 Spectrophotometer (Thermofisher, Waltham, MA). cDNA was synthesized using iScript™ cDNA Synthesis Kit (Bio-Rad), and qPCR was performed using SsoAdvanced™ Universal SYBR® Green Supermix (Bio-Rad) in the Bio-Rad CFX96 Touch thermocycler using the following conditions: 95°C 3 min, 1 cycle; followed by (95°C 10sec, 55°C 30sec), 39 cycles. Specificity of qPCR reactions was assessed using melt curve analysis (60°C to 95°C, 0.5°C increments). Reference genes were selected by testing stability of expression (M-score) of common housekeeping mRNAs in a pre-designed reference gene plate (Reference genes H96, Bio-Rad) using cDNA from control. M-score analysis identified PSMC4, PUM1 and IPO8 as transcripts with highest stability between our conditions. The geometric mean of these three reference genes was used for normalization. qPCR primers were designed using the IDT PrimerQuest® tool; primer sequences were as follows: integrin α3, Fwd-GCAGGTAATCCATGGAGAGAAG, Rev-CCACTAGAAGGTCTGGGTAGAA; PSMC4, Fwd-GGAGGTTGACTTGGAAGACTATG, Rev-GACAGCCAACATTCCACTCT; PUM1, Fwd-TACGTGGTCCAGAAGATGATTG, Rev-GCCATAGGTGTACTTACGAAGAG; IPO8, Fwd-CATGATGCCTCTCCTGCATAA, Rev-CTTCTCCTGCATCTCCACATAG. Melt curves and Ct values were accessed using Bio-Rad CFX Manager software.

### Flow cytometry

Cells were trypsinized then blocked in suspension with 10% goat serum/PBS, then incubated with 5 μg/ml anti-α3 integrin monoclonal antibody P1B5 (MAB1952Z; EMD Millipore Corp, Burlington, MA) or normal mouse IgG as control (sc-2025; Santa Cruz Biotechnology), followed by secondary antibody, allophycocyanin, crosslinked, goat anti-mouse IgG (Invitrogen) (1:200 dilution). Flow cytometry was performed on a FACSCalibur (Becton Dickinson, Franklin Lakes, NJ), and data were analyzed using the FlowJo software (Becton Dickinson).

### Experimental metastasis assay

Experimental metastasis assays were performed as described previously [12, 49]. Briefly, cells were labeled fluorescently by stable transduction with a lentivirus that expresses ZsGreen (pHAGE-IRES-ZsGreen), as described [12]. For each variant, 1x10^4^ cells were injected into the tail veins of 5-week old female NSG™ mice (Jackson Laboratories, stock# 005557: NOD.Cg-Prkdc<scid>II2rg<tm1Wjl>SzJ, Bar Harbor, ME). After 21 weeks lungs were harvested and imaged using a Leica M205 FA & Lecia DCF3000 G (Leica Microsystems, Wetzlar, Germany). Lung metastases were counted manually on both sides of all lung lobes and totaled for each mouse. Animal experiments were approved by the Institutional Animal Care and Use committee at Albany Medical College.

### Matrigel invasion assay

Transwell invasion chambers (8 μM pore filter; Corning) were coated with 400 μg/mL Matrigel (Fisher Scientific, Waltham, MA) then incubated overnight at 37°C. A total of 8x10^4^ cells were seeded in complete growth medium into the upper chamber, and growth medium supplemented with 20% fetal bovine serum was placed in the lower chamber as chemoattractant. Plates were incubated at 37°C for 18 hours to allow cells to invade through the Matrigel layer, and cotton swabs were used to remove non-invading cells from the top sides of filters. The bottom sides of filters were then fixed with 100% ice-cold methanol and stained with 40,6-Diamidino-2-Phenylindole, Dihydrochloride (DAPI) to visualize nuclei. Images of three random fields per chamber were obtained using a Nikon eclipse TE2000-U inverted microscope (Nikon Microscopy, Minoto city, Tokyo, Japan), and the number of invaded cells was quantified using Fiji imageJ. Cell invasion was quantified from 3 independent experiments, wherein each condition was plated in duplicate.

### RNA-seq

Pellets of 1x10^6^ cells were collected and the RNA isolation, library construction for RNAs and the sequencing were performed by GENEWIZ (South Plainfield, NJ). Scatterplots of pairwise comparisons of RNA-seq data were generated using ExAtlas (https://lgsun.irp.nia.nih.gov/exatlas/index.html) with FDR threshold of 0.05 and fold change threshold of 2.0. RNA-seq datasets related to this article have been deposited in the Harvard Dataverse (https://dataverse.harvard.edu/) with the following doi: https://doi.org/10.7910/DVN/D9G2GQ. Gene ontology analysis was provided by GENEWIZ.
